# Sugar-Mediated Green Synthesis of Silver Selenide Semiconductor Nanocrystals under Ultrasound Irradiation

**DOI:** 10.3390/molecules25215193

**Published:** 2020-11-08

**Authors:** Daniela Armijo García, Lupe Mendoza, Karla Vizuete, Alexis Debut, Marbel Torres Arias, Alex Gavilanes, Thibault Terencio, Edward Ávila, Clayton Jeffryes, Si Amar Dahoumane

**Affiliations:** 1 School of Biological Sciences and Engineering, Yachay Tech, San Miguel de Urcuquí 100650, Ecuador; lupe.mendoza@yachaytech.edu.ec; 2 Center of Nanoscience and Nanotechnology, Universidad de las Fuerzas Armadas ESPE, Sangolquí 171103, Ecuador; ksvizuete@gmail.com (K.V.); apdebut@espe.edu.ec (A.D.); mmtorres@espe.edu.ec (M.T.A.); alexgavilanes1993@gmail.com (A.G.); 3 School of Chemical Sciences and Engineering, Yachay Tech, San Miguel de Urcuquí 100650, Ecuador; tthibault@yachaytech.edu.ec (T.T.); eavila@yachaytech.edu.ec (E.Á.); 4 Center for Advances in Water and Air Quality & The Dan F. Smith Department of Chemical & Biomolecular Engineering, Lamar University, Beaumont, TX 77710, USA; cjeffryes@lamar.edu

**Keywords:** green chemistry, silver selenide, nanoparticles, semiconductors, fructose, starch, in vitro toxicity, sonochemistry

## Abstract

Silver selenide (Ag_2_Se) is a promising nanomaterial due to its outstanding optoelectronic properties and countless bio-applications. To the best of our knowledge, we report, for the first time, a simple and easy method for the ultrasound-assisted synthesis of Ag_2_Se nanoparticles (NPs) by mixing aqueous solutions of silver nitrate (AgNO_3_) and selenous acid (H_2_SeO_3_) that act as Ag and Se sources, respectively, in the presence of dissolved fructose and starch that act as reducing and stabilizing agents, respectively. The concentrations of mono- and polysaccharides were screened to determine their effect on the size, shape and colloidal stability of the as-synthesized Ag_2_Se NPs which, in turn, impact the optical properties of these NPs. The morphology of the as-synthesized Ag_2_Se NPs was characterized by transmission electron microscopy (TEM) and both α- and β-phases of Ag_2_Se were determined by X-ray diffraction (XRD). The optical properties of Ag_2_Se were studied using UV–Vis spectroscopy and its elemental composition was determined non-destructively using scanning electron microscopy–energy-dispersive spectroscopy (SEM–EDS). The biological activity of the Ag_2_Se NPs was assessed using cytotoxic and bactericidal approaches. Our findings pave the way to the cost-effective, fast and scalable production of valuable Ag_2_Se NPs that may be utilized in numerous fields.

## 1. Introduction

Semiconductor materials are technologically important due to their size-dependent optical, structural and electrical properties [1,2]. Phase-change chalcogenides have the ability to alternate between amorphous and crystalline phases with exposure to controlled energy inputs, making them an important material for a wide range of optical and electronic applications [3,4,5,6]. Silver selenide (Ag_2_Se), also known as naumannite, is a semiconductor material as a bulk and rarely found in nature as a mineral. It belongs to I-VI compounds with an optical band gap of 1.2–1.8 eV [7]. Among semiconductor nanomaterials, Ag_2_Se is one of the most widely investigated chalcogenide nanomaterials due to its countless applications, for instance, in electronics and the biomedical field [8,9]. Silver selenide is a mixed ionic conductor with a phase transition at atmospheric pressure from a low-temperature orthorhombic phase (β-Ag_2_Se), with a narrow direct band gap predicted by simulation to be 0.07–0.15 eV at 0 K, to a high-temperature cubic phase (α-Ag_2_Se, >407 K) [10]. Orthorhombic β-Ag_2_Se is the most accepted crystal structure of Ag_2_Se due to its relatively high Seebeck coefficient (thermoelectric power, −150 μV K^−1^) at 300 K and an unusually low lattice thermal conductivity coupled with high electrical conductivity [11]. β-Ag_2_Se exhibits both photocatalytic activity and fluorescence. This compound has been used as a photosensitizer in photographic films, thermo-chromic materials for nonlinear optical devices and photovoltaic cells [12].

Conventionally, Ag_2_Se nanoparticles (NPs) are produced by different methods, such as chemical conversion [13], hydrothermal reaction [14,15] and hot injection [16], while their microstructured analogs might be fabricated via, for instance, electrodeposition [17] and thermal evaporation [17]. However, these complicated and expensive methods involve both toxic chemicals and extreme conditions, generating hazardous toxic byproducts. As an alternative solution to this concern, “green chemistry” provides 12 principles as a guide in implementing less harmful synthesis methods [18].

Bioprocesses employing natural reagents, plant materials, microorganisms or their extracts have proven efficient in the synthesis of inorganic NPs, usually in a nontoxic, aqueous medium [19,20,21]. The green synthesis of NPs offers advantages over the traditional physical and chemical methods. In addition to working at mild temperatures, there is no need for high-pressure equipment. Moreover, the cost of disposing of solvents and chemical waste is significantly reduced. Overall, green synthesis is simple to carry out and cost-effective as it relies on renewable natural resources and results in more compatible and stable products [18].

Several routes have been devised in green nanotechnology, including biological methods [18], ultrasound (US) energy [22,23,24], microwave irradiation [25,26,27,28] or the use of Tollens’ reagent [29,30] for the synthesis of valuable NPs of controlled shapes and sizes. However, nanoparticles are naturally unstable and tend to aggregate due to the onset of strong van der Waals forces [31]. Capping agents protect and passivate the particle surface preventing their agglomeration, as well as their uncontrolled growth [32]. Among the materials used as stabilizers, natural polymers and biomolecules, such as nucleic acids, lipids and proteins, provide an excellent steric hindrance effect along with potent stability [33,34,35,36].

Fructose, the monosaccharide used in this study, is a nontoxic reducing agent and constitutes a cheap and abundant natural source for the synthesis of inorganic NPs, such as AuNPs [37]. This reducing character is achieved in its open-chain form and maximized with its ionization [37]. In combination, reducing sugars and stabilizing biopolymers can mediate the formation of stable, morphologically controlled NPs.

Green approaches are also used to produce nontoxic and biocompatible nanoparticles for unique applications in industry [38] and biomedicine such as antibacterial, antifungal and antiviral agents [39,40,41]. Moreover, advances in modern medicine are driven by an increased use of NPs as efficient drug delivery vectors and powerful antimicrobial weapons [42,43]. Generally, inappropriate use and abuse of antibiotics are the main cause of induced antibiotic resistance in bacteria. Antibiotic-resistant bacterial infections may lead to high drug doses, higher toxicity treatments, prolonged hospitalization periods and an increase in morbidity and mortality [44].

In this context, the present article describes, for the first time, the sustainable production of silver selenide nanoparticles (Ag_2_Se NPs) via a simple one-step ultrasound-assisted method by mixing aqueous solutions of silver nitrate (AgNO_3_) and selenous acid (H_2_SeO_3_) that act as Ag and Se sources, respectively. D-fructose is used as the reducing and coprecipitating agent for the formation of Ag_2_Se NPs, replacing toxic and harmful agents. On the other hand, starch acts as the stabilizing agent that controls the growth of Ag_2_Se NPs. Finally, the biological activity of these ecofriendly-synthesized Ag_2_Se NPs is assessed from cytotoxic and bactericidal points of view.

## 2. Results and Discussion

### 2.1. Visual Aspect of Ag_2_Se NPs

**Figure 1:**
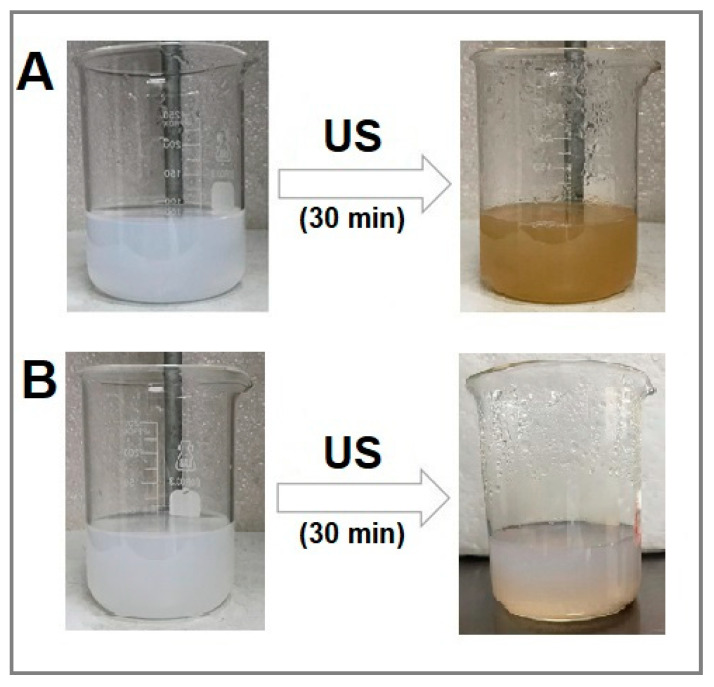
Pathway for the synthesis of silver selenide nanoparticles (NPs). (**A**) Sample made of 3.4 mM [Ag^+^]_f_ and 1.7 mM [Se^4+^]_f_ in the presence of 40 mg mL^−1^ of fructose and 10 mg mL^−1^ of starch exposed to ultrasound (US) irradiation for 30 min (59-s pulse ON, 5-s pulse OFF). (**B**) Control sample made of 3.4 mM [Ag^+^]_f_ and 1.7 mM [Se^4+^]_f_ exposed to US irradiation under the same conditions as in (**A**).

The initial observation of the formation of Ag_2_Se NPs consists of a visual color change of the samples after ultrasound (US) irradiation. The results clearly show that the reaction mixture containing silver and selenium precursors, in addition to fructose and starch as the reducing and stabilizing agents, respectively, turns from white to brown after US irradiation (Figure 1A). The US irradiated control containing only 3.4 mM [Ag^+^]_f_ and 1.7 mM [Se^4+^]_f_ aqueous solutions showed no significant change in color (Figure 1B), confirming the effectiveness of fructose and starch as green agents when combined with US energy to promote the production of Ag_2_Se NPs.

### 2.2. UV–Vis Spectroscopy

**Figure 2:**
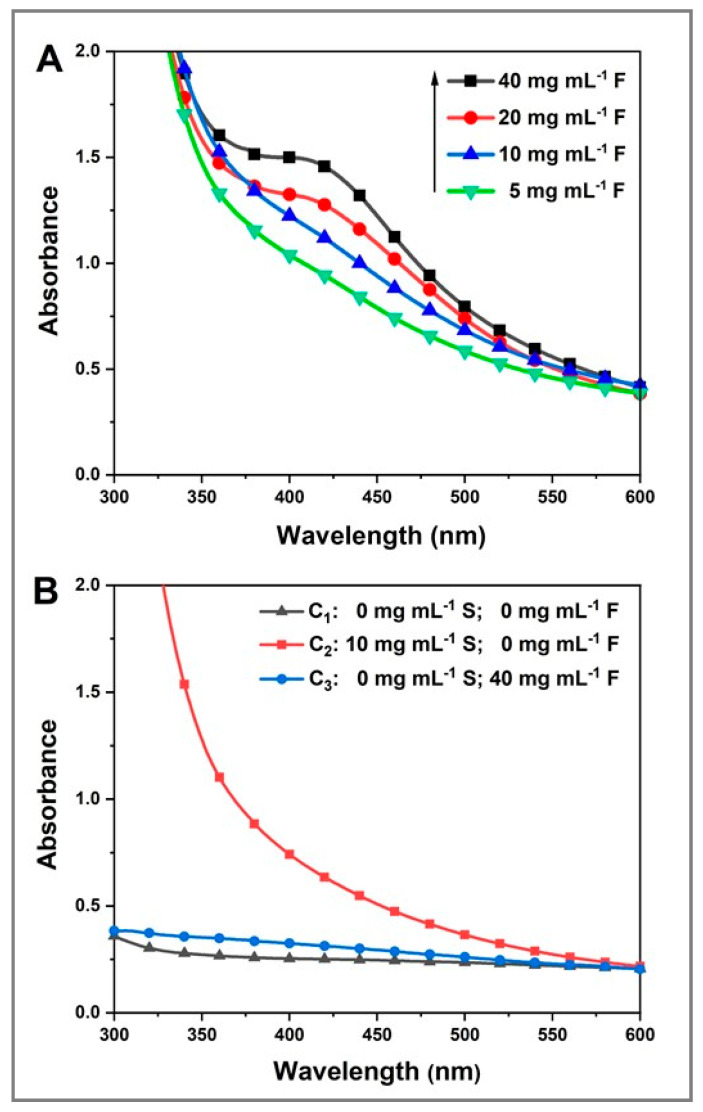
UV–Vis spectra of the as-synthesized Ag_2_Se NPs; (**A**) at different fructose concentrations and a constant starch concentration of 10 mg mL^−1^; and (**B**) control experiments. **F:** fructose; **S:** starch.

The formation of Ag_2_Se NPs was confirmed by the appearance of a unique and well-defined absorption band between 385 and 446 nm with a maximum at ~413 nm, similar to that reported in the literature [45,46] (Figure 2A). In this range, the single absorption peak gives information about the shape, size and stability/aggregation of the NPs [47,48].

It is notable that the intensity of the absorption band of the as-obtained Ag_2_Se NPs increases as the fructose concentration increases from 5 to 40 mg mL^−1^, while the other experimental parameters are kept constant (Figure 2A). This confirms the crucial role played by fructose in Ag_2_Se NP formation. Hypothetically, this process may consist of two steps: first, cationic selenium is reduced to Se^2−^ that, in turn, coprecipitates with cationic silver (Ag^+^) to subsequently give rise to Ag_2_Se NPs. The variations in the absorption intensities indicate significant differences in the particle number between the samples [49]. On the other hand, the corresponding UV–Vis absorption spectra of the control experiments C_1_, C_2_ and C_3_ exhibit no absorption band (Figure 2B). This denotes either the absence of any Ag_2_Se NP formation or the instability of any formed Ag_2_Se NPs.

**Figure 3:**
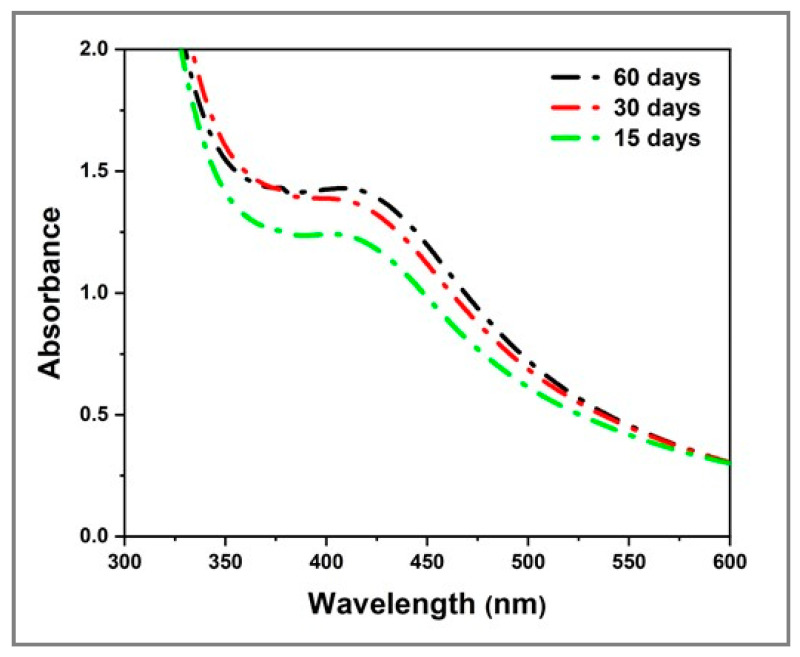
UV–Vis spectra of Ag_2_Se NPs obtained from sample E_4_ (3.4 mM [Ag^+^]_f_, 1.7 mM [Se^4+^]_f_, 40 mg mL^−1^ fructose and 10 mg mL^−1^ starch) stored at RT in the dark recorded at 15, 30 and 60 days.

After confirming their production and the key role of fructose in this reaction, the colloidal stability of the Ag_2_Se NPs obtained from sample E_4_, made of 3.4 mM [Ag^+^]_f_, 1.7 mM [Se^4+^]_f_, 40 mg mL^−1^ fructose and 10 mg mL^−1^ starch and stored at room temperature (RT) in the dark, was monitored by recording its UV–Vis spectra at 15, 30 and 60 days (Figure 3). As a result, the absorption bands display similar features although a slight increase in the intensity after 30 and 60 days is noticed. This slight increase in the absorbance intensity might be attributed to either experimental errors or reorganization of the polysaccharidic matrix that surrounds and stabilizes the NPs. Furthermore, no signs of sedimentation were observed [50]. These findings confirm the stability of the as-produced Ag_2_Se NPs over a long period of time.

**Figure 4:**
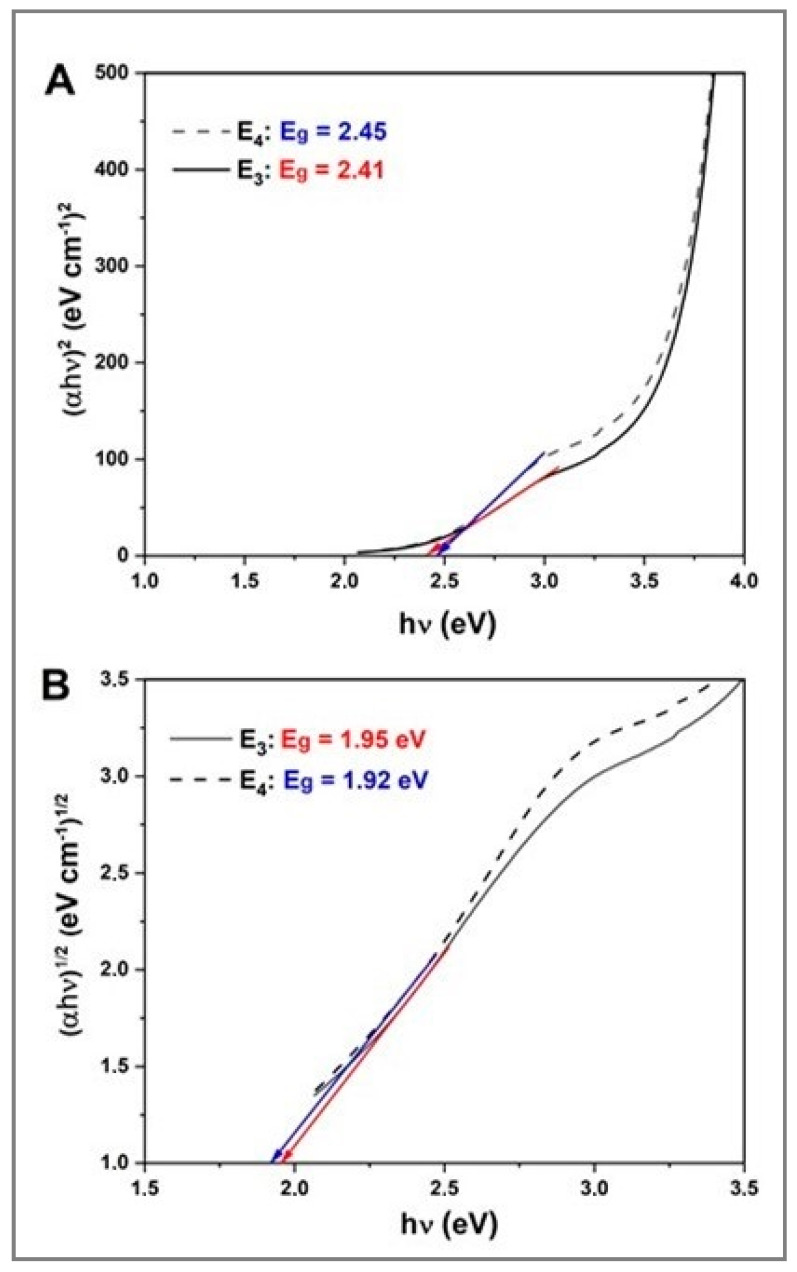
Tauc plot of (αhν)^1/2^ vs. (hν) of Ag_2_Se NPs obtained from the samples: (**A**) E_3_ (3.4 mM [Ag^+^]_f_, 1.7 mM [Se^4+^]_f_, 20 mg mL^−1^ fructose, 10 mg mL^−1^ starch), and (**B**) E_4_ (3.4 mM [Ag^+^]_f_, 1.7 mM [Se^4+^]_f_, 40 mg mL^−1^ fructose, 10 mg mL^−1^ starch).

Figure 4 displays the Tauc plots, i.e., (*αhν*)*^*1*/n^* vs. (*hν*)^2^ of the Ag_2_Se NPs obtained from samples E_3_ (3.4 mM [Ag^+^]_f_, 1.7 mM [Se^4+^]_f_, 20 mg mL^−1^ fructose, 10 mg mL^−1^ starch) and E_4_ (3.4 mM [Ag^+^]_f_, 1.7 mM [Se^4+^]_f_, 40 mg mL^−1^ fructose, 10 mg mL^−1^ starch). The plots reveal that the Ag_2_Se NPs undergo both direct and indirect transitions. In this particular case, the direct transition (Figure 4A) seems to have two linear segments with a possible second band gap that could come from the two phases determined in XRD (vide infra) and/or may be due to the presence of a bimodal NP population as observed with TEM (vide infra). The occurrence of both direct and indirect transitions was also previously reported for Ag_2_S NPs and Ag_2_Se NPs [51]. The indirect band gaps of samples E_3_ and E_4_, displayed in Figure 4B, were found to be 1.95 and 1.92 eV, respectively, which suggests optical absorption at wavelengths below 635 and 645 nm, respectively. The direct band gaps of the same samples were found to be 2.41 and 2.45 eV, respectively (Figure 4A). In other words, there is no significant difference between these two band gap values, therefore the amount of fructose does not seem to have any impact on the band gap of the as-produced Ag_2_Se NPs. However, the band gap values of these Ag_2_Se NPs are higher than the ones experimentally estimated for thin films of the same material (1.37 eV) [52] or for their bulk counterpart (0.16 eV) [11], suggesting that it is possible to modify the band gap by tailoring the NP size. The difference between the reported values and the obtained *E_g_* can be attributed to the appearance of a series of discrete states in the conduction and valence bands yielding an *E_g_* increase in quantum size systems [53,54].

### 2.3. X-ray Diffraction

**Figure 5:**
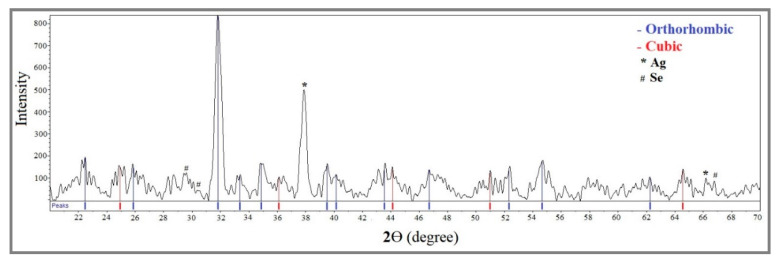
XRD diffractogram of Ag_2_Se NPs from sample E_3_. The red and blue vertical lines correspond to the cubic and orthorhombic phases of Ag_2_Se, respectively.

Ag_2_Se NPs can exist as either an orthorhombic β-phase or a cubic α-phase. According to Kumashiro et al. [55], Ag_2_Se is a non-stoichiometric compound in both phases that can co-exist with Ag_2_Se as a single phase. In addition, silver solubility is higher in the α-phase than in the β-phase. Figure 5 displays the XRD pattern of Ag_2_Se NPs obtained from sample E_3_ made of 3.4 mM [Ag^+^]_f_ and 1.7 mM [Se^4+^]_f_ in the presence of 20 mg mL^−1^ fructose and 10 mg mL^−1^ starch. The following characteristic diffraction peaks are observed at 2θ = 36.1° (002), 44.1° (112), 52.3° (022) and 64.5° (222), corresponding to planes of the cubic α-Ag_2_Se phase (JCPDS card no. 98-003-3627). The additional diffraction peaks observed at 2θ = 25.8°, 31.8°, 33.3°, 34.8°, 39.5°, 40.1°, 43.5°, 46.6°, 50.9° and 54.6° correspond to the (012), (102), (112), (121), (031), (122), (210), (004), (221) and (222) planes, respectively, which can be assigned to the orthorhombic β-Ag_2_Se phase (JCPDS card no. 98-001-5213) corresponding to the naumannite structure. Additionally, the prominent peak at 2θ = 37.6° suggests the presence of elemental silver and might correspond to the (111) plane of the face-centered cubic (fcc) structure of metallic silver (JCPDS file no. 03–0921). Furthermore, weak peaks can be attributed to selenium particles. These last two observations suggest that the precursor cations were reduced to their elemental forms in the final sample.

The presence of both orthorhombic and cubic phases of silver selenide is due to the acoustic cavitation phenomenon which causes the formation of vapor bubbles that, after collapsing, produce localized hot spots with temperatures up to 5000 K, high pressures up to 20 MPa and very high cooling rates [56]. In other words, this method offers optimal conditions for the simultaneous formation of both phases. Furthermore, widening appears in some of the diffraction peaks, mainly due to the small particle size as confirmed by TEM micrographs (vide infra) which yields peak overlapping of the two phases.

The average crystallite size of the as-produced Ag_2_Se NPs, determined from the half-width of the diffraction peak at 2θ = 31.8° corresponding to the (102) crystallographic plane of the β-phase using the Debye–Scherrer formula (Equation (2)), was found to be 26.5 nm, in very good agreement with the size range of the nanoparticles (5–40 nm) deduced from the TEM micrographs (vide infra). This result is consistent with the size of the crystallite and similar phases previously reported in the literature [52,54,56].

### 2.4. Transmission Electron Microscopy

**Figure 6:**
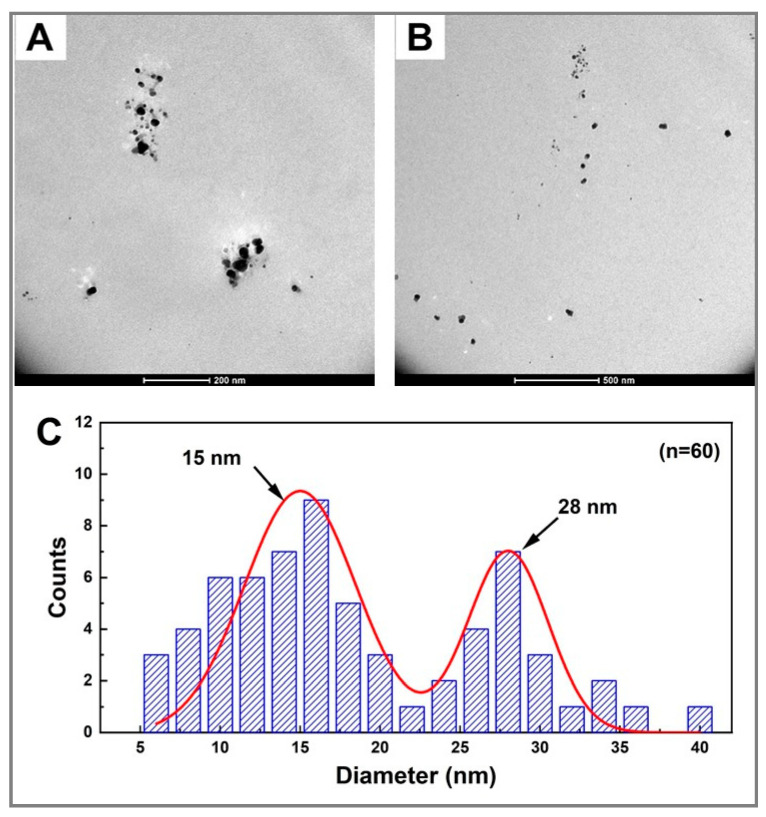
TEM micrographs of colloidal Ag_2_Se NPs from sample E_3_. (**A**,**B**) TEM images at different magnifications where the NPs are the black spots, and (**C**) histogram plot with the average size values of Ag_2_Se NPs (*n* = 60).

TEM images revealed that the as-synthesized Ag_2_Se NPs from sample E_3_ (3.4 mM [Ag^+^]_f_, 1.7 mM [Se^4+^]_f_, 20 mg mL^−1^ fructose, 10 mg mL^−1^ starch) are round in shape, well-dispersed and well-defined with a broad size distribution ranging between 5 and 40 nm (Figure 6A,B). Figure 6C depicts the corresponding mean particle size distribution of the same sample. It shows the presence of a heterogeneous population of Ag_2_Se NPs with a bimodal size distribution. The average particle sizes estimated from TEM images with a population of 60 NPs were found to be 15 and 28 nm, which could be due to a non-homogeneous reaction medium in terms of temperature, matter composition and processing time. In fact, the longer the ultrasonic radiation treatment, the higher the energy contribution. This triggers a greater cavitation effect that generates higher temperatures resulting in smaller mean NP diameters [57].

**Figure 7:**
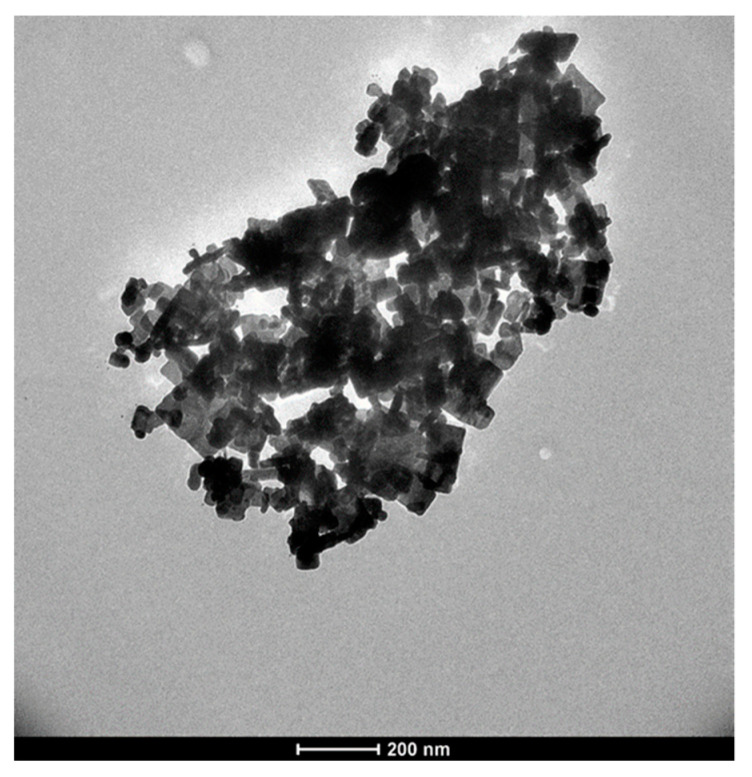
TEM image of Ag_2_Se NPs from sample C_2_ prepared in the absence of fructose.

Although no UV–Vis absorption band was recorded for the fructose-free control sample C_2_ (3.4 mM [Ag^+^]_f_, 1.7 mM [Se^4+^]_f_, 10 mg mL^−1^ starch) (Figure 2B), the TEM micrograph of the same sample displays particles whose morphology varies from spherical to cubic and rod-shaped nanocrystals with a large particle size (Figure 7), as compared to Figure 6. Moreover, large aggregate nanostructures were formed; however, their chemical nature was not determined. This might be attributed to the fact that starch, a natural polysaccharide, can be decomposed into amylose and amylopectin, natural polymers formed by long chains of α-D-glucose molecules. α-D-glucose, like D-fructose, is a natural sugar that acts as a reducing agent in the production of inorganic NPs [58]. Therefore, starch, after its possible degradation, might have partially acted as the reducing agent instead of the stabilizing agent in the presence of fructose. Consequently, aggregated cubic and rod NPs of different sizes were formed since there is little to no stabilizing matrix to control the NP features (size, shape, stability), corroborating the absence of any UV–Vis absorption band. These results confirm the key roles of fructose as a strong reducing sugar and starch as an efficient stabilizing matrix in the ultrasound-assisted production of nanoparticles with interesting optical properties and controlled morphological features.

### 2.5. Scanning Electron Microscopy–Energy-Dispersive Spectroscopy

**Figure 8:**
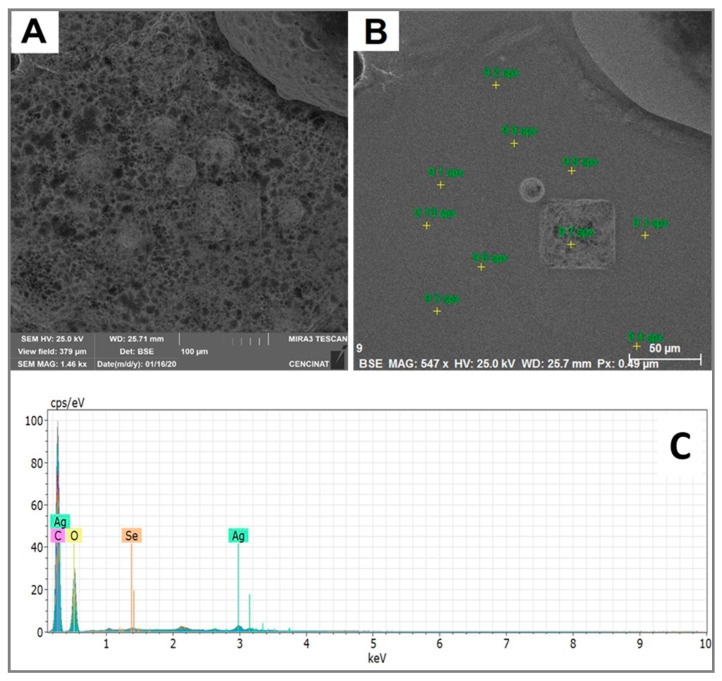
(**A**) SEM image; (**B**) selected areas for EDS spot analysis; and (**C**) EDS representative spectrum of Ag_2_Se NPs.

SEM–EDS was used to study the chemical composition and purity of the as-obtained Ag_2_Se nanomaterial. The EDS spectrum, taken from selected areas of SEM images, exhibits prominent silver, selenium, carbon and oxygen peaks in the as-synthesized Ag_2_Se NPs (Figure 8). The detected C and O mainly arise from the stabilizing agent (starch) and the carbon grid used to mount the sample. The identification line for the major emission energy of selenium was observed in the range of 1.3–1.4 keV, while silver was observed in the range of 2.8–3.0 keV, corresponding in both cases to the L-α peak of these elements. These selenium and silver peaks, due to their presence in Ag_2_Se NPs, are in good agreement with elemental peaks reported in the literature [45,59]. Therefore, the EDS spectrum confirms the presence of silver and selenium atoms in the as-synthesized Ag_2_Se NPs. Moreover, by dividing the peak intensity of Ag by the one of Se, this results in a 2:1 ratio, suggesting the chemical formula Ag_2_Se.

### 2.6. Biological Activity

#### 2.6.1. Cytotoxicity Assessment by MTT Assay

The cytotoxicity of the as-synthesized Ag_2_Se NPs was evaluated using the MTT assay considering that viability assays are key to explaining the cellular response to a toxic agent and provide information about the cell survival and metabolic activity. Human fibroblasts from the ATCC cell line were treated with different concentrations of Ag_2_Se NPs for 24, 48 and 72 h. The obtained results indicated a range of cytotoxicity responses when compared to the control groups.

**Figure 9:**
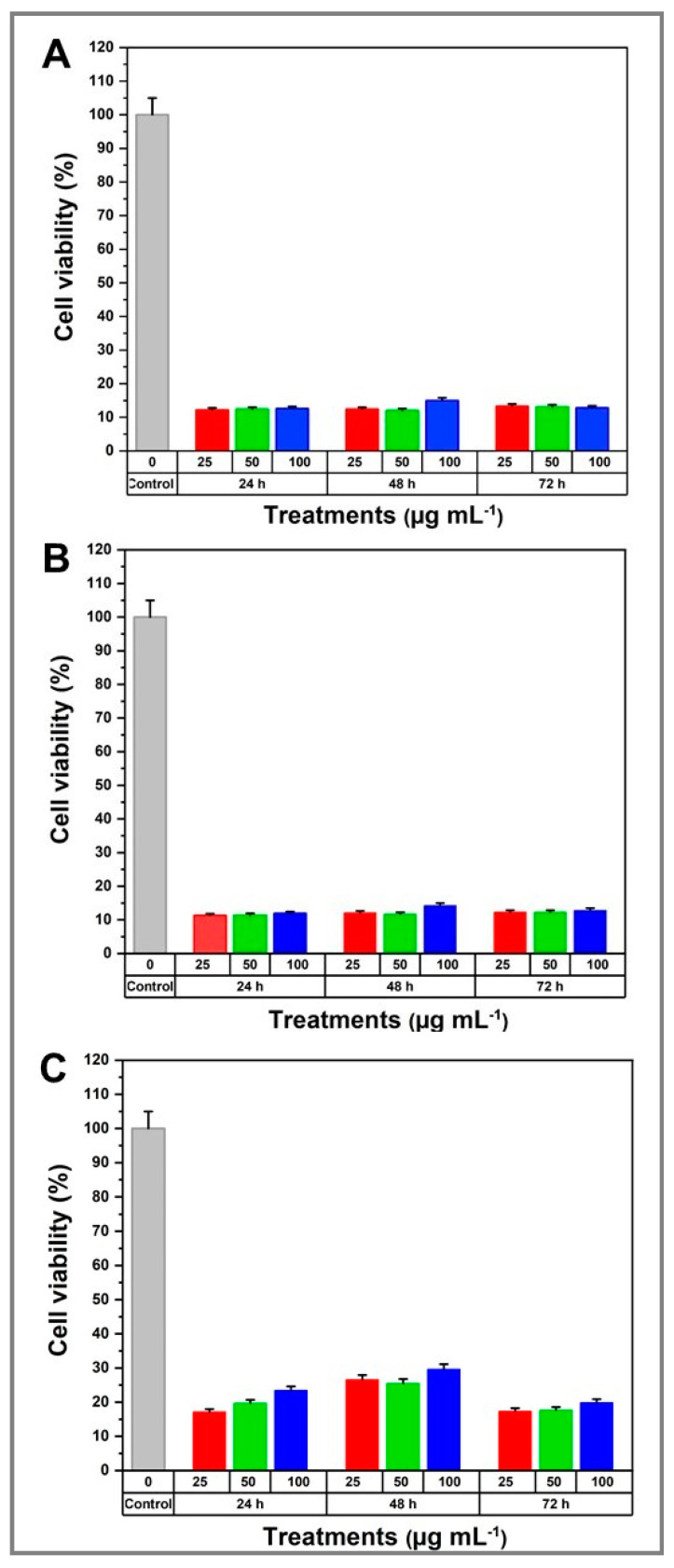
Cell viability of ATCC human fibroblasts exposed to Ag_2_Se NPs at 0, 25, 50 and 100 µg mL^−1^ concentrations for 24, 48 and 72 h. (**A**) Sample E_1_; (**B**) sample E_2_; and (**C**) sample E_3_.

The as-synthesized Ag_2_Se NPs caused a significant reduction in the mitochondrial activity of ATCC cells with respect to the untreated control group (Figure 9). For instance, cultures at the studied NP concentrations of the E_3_ sample (3.4 mM [Ag^+^]_f_, 1.7 mM [Se^4+^]_f_, 10 mg mL^−1^ starch, 20 mg mL^−1^ fructose) showed a decrease in the cytotoxic effect with respect to the controls, reaching values close to ~30% of viability after incubation for 24 and 48 h, following a concentration-dependent effect (Figure 9C). After 72 h post-NP exposure, the cytotoxicity slightly increased as the viability decreased to ~20%, regardless of the lyophilized Ag_2_Se NP concentration. On the other hand, samples E_1_ and E_2_ exhibited a higher cytotoxicity (~10% cell viability) that was independent of the Ag_2_Se NP concentration and exposure time (Figure 9A,B). According to these results, the as-synthesized Ag_2_Se NPs display a very strong biocidal activity against ATCC human fibroblast cell cultures. Moreover, the initial amount of fructose may impact this activity.

#### 2.6.2. Bactericidal Tests

The antibacterial activity of Ag_2_Se NPs was investigated against the following pathogenic microorganisms by the disc diffusion method: *Escherichia coli*, *Staphylococcus aureus*, *Salmonella typhimurium* and *Pseudomonas aeruginosa*. The maximum zone of inhibition (MZI), summarized in Table 1, does not exhibit any trend related to the bacterial cell wall type. On the other hand, there seems to be a trend among the Gram-species. Indeed, the Ag_2_Se NPs are very toxic towards *E. coli*, moderately toxic towards *S. typhimurium* and have the lowest toxicity to *P. aeruginosa*. Moreover, Ag_2_Se NPs exhibit a very high toxicity against the Gram + *S. aureus*. Additionally, the initial amount of fructose, the only difference in the composition of E_1_, E_2_ and E_3_ samples, does not seem to impact the bactericidal activity of the as-obtained Ag_2_Se NPs. Furthermore, the increase in lyophilized Ag_2_Se NP concentration expands the MZI for all the pathogenic bacteria (results not shown).

The mechanism of the Ag_2_Se NP bactericidal mode of action might differ according to the bacterial species and the composition of the samples. Gram − and Gram + bacteria show a similar antibiotic effect but at different sample compositions, as seen in the case of *P. aeruginosa*. In fact, only two Ag_2_Se NP samples, E_1_ and E_3_, at their maximum concentration showed a slight bactericidal effect, whereas all samples promoted a strong antibiotic effect at all NP concentrations against *E. coli*.

Although the antimicrobial mechanism of semiconductor NPs is not entirely understood, studies suggest this might be related to the formation of oxidative stress in cells, causing damage to their biomolecules that yields cell death [2]. In the present study, it is possible that Ag_2_Se NPs penetrate the cell membrane and irreversibly damage its antioxidative systems in a concentration-dependent manner.

## 3. Materials and Methods

### 3.1. Materials

For the preparation of the colloidal silver selenide NPs, selenous acid, H_2_SeO_3_ (99%, Sigma-Aldrich, St. Louis, MO, USA), and silver nitrate, AgNO_3_ (99%, Emsure, Darmstadt, Germany), were the precursors, D(+) fructose (Sigma-Aldrich, St. Louis, MO, USA) was the reducing agent, soluble corn starch (Sigma-Aldrich, St. Louis, MO, USA) was the stabilizing agent and deionized water (DIW) was the solvent. All chemicals were used without any additional chemical purification.

### 3.2. Green Synthesis of Silver Selenide NPs

The green synthesis of Ag_2_Se NPs was carried out via a facile co-precipitation method. Selenous acid (0.44 g) and AgNO_3_ (1.73 g) were separately dissolved in DIW at room temperature (RT). The preparation of Ag_2_Se NPs and the associated control experiments are described in Table 2. For Ag_2_Se NP preparation, 5 mL of 34 mM H_2_SeO_3_ and 10 mL of 34 mM AgNO_3_ were added into a 300 mL glass beaker. Immediately after, varying masses of starch and fructose were added to the reaction mixture and the total volume was brought to 100 mL by adding DIW. Finally, ultrasound irradiation was carried out using a Cole-Parmer ultrasonic processor (DAIGGER GE 505, 500 W, 20 kHz, Daigger Scientific, Buffalo Grove, IL, USA) whose probe was directly immersed into the reaction medium. The operating condition was 59-s pulse ON and 5-s pulse OFF with an amplitude of 70% for 30 min. The formation of Ag_2_Se NPs was confirmed by a color change in the reaction mixture.

### 3.3. Physico-Chemical Characterization

#### 3.3.1. Ultraviolet–Visible (UV–Vis) Spectroscopy

The absorption characteristics of the as-obtained Ag_2_Se NPs and their colloidal stability over time were determined by recording the UV–Vis spectra from 300 to 600 nm using a LAMBDA 1050 UV-Vis Spectrophotometer (Perkin Elmer, Inc., Shelton, CT, USA). All measurements were performed in a quartz cuvette, using DIW as the blank. Subsequently, all the samples were stored at RT in the dark.

Starting from the UV–Vis spectra of samples E_3_ and E_4_, samples with fructose concentrations of 20 and 40 mg L^−1^, respectively, the band gap of the as-produced Ag_2_Se NPs was determined using a Tauc plot relating the absorption coefficient (α) and the photon energy (*hν*), as depicted in Equation (1):

α*hν* = *A*(*hν* − *E_g_*)*^*1*/n^* (1)

where *E_g_* is the band gap in eV, *h* is Planck’s constant (6.626 10–34 J s^−1^), *ν* is the frequency in Hz, *A* and *α* are constants and “*n*” is 1/2, 3/2, 2 or 3 for direct allowed, direct forbidden, indirect allowed and indirect forbidden transitions, respectively. The extrapolation of the linear portion of the curve with the *x*-axis gives the value of the *E_g_*.

#### 3.3.2. X-ray Diffraction (XRD)

XRD (PANanalytical, EMPYREAN, Almelo, The Netherlands) analysis on lyophilized Ag_2_Se NPs was carried out to identify the phase structure and determine the crystallite size using a θ–2θ configuration (generator–detector), with the Cu-Kα excitation source at λ = 1.54059 Å.

Additionally, the broadening of the diffraction peak in the XRD is related to the particle size by the Debye–Scherrer formula (Equation (2)) to obtain the average crystallite size:

L = Κλ/Bcosθ(2)

where λ is the X-ray wavelength (0.154059 nm), B is the line broadening at full width half maximum (FWHM), θ is the diffraction angle, K is the constant related to crystalline shape (0.93 for spherical NPs) and L is the linear dimension of the particle.

#### 3.3.3. Transmission Electron Microscopy (TEM)

TEM (FEI, TECNAI, G2spirit twin, Holland) was employed to determine the size, shape and morphologies of the as-produced Ag_2_Se NPs. The samples were prepared by casting one drop of Ag_2_Se NP solution onto a carbon-coated copper grid and dried in ambient air at RT.

TEM micrographs at 200 and 500 nm scales were subjected to particle size distribution analyses using ImageJ software (developed by the National Institutes of Health (NIH), Bethesda, MD, USA) to generate. CSV files with information about the cross-sectional area of the as-synthesized nanoparticles. As Ag_2_Se NPs are spherical, the diameter was calculated from the area (A = πD^2^/4). Then, using OriginPro software, the size distribution histogram of 2 nm resolution was generated and fitted with a Gaussian function to determine the average size of the NPs.

#### 3.3.4. Scanning Electron Microscopy–Energy-Dispersive Spectroscopy (SEM–EDS)

The elemental analysis and mapping of Ag_2_Se NPs were performed non-destructively using SEM (TESCAN, MIRA 3, Brno, Czech Republic) equipped with EDS (Bruker Nano GmbH, Quantax, Berlin, Germany). The samples were prepared by casting one drop of the Ag_2_Se NP reaction media onto a stub previously covered with two layers of double-coated carbon conductive tape and dried in ambient air at RT.

### 3.4. Biological Activity of Ag_2_Se NPs

#### 3.4.1. MTT Assay Using Human Fibroblasts

The ATCC human fibroblast (HFF) cell line was grown in DMEM medium supplemented with 10% wt/v fetal bovine serum (FBS) and 1% wt/v penicillin/streptomycin. Cells were incubated at 37 °C in a humidified 5% CO_2_ atmosphere. Subsequently, the cells were harvested using 0.25% wt/v trypsin-EDTA (1X) and the cell count was performed using trypan blue and Neubauer chamber. An amount of 100 µL of the cell suspension was dispersed to achieve 10^4^ cells/well in 96-well culture plates and incubated under the same conditions for another 24 h. Then, the medium was replaced with a medium containing the lyophilized Ag_2_Se NPs at different concentrations: 0 (control), 25, 50 and 100 µg mL^−1^. After 24, 48 and 72 h incubation, the MTT solution at 0.5 mg mL^−1^ was added to each well and incubated for another 4 h at 37 °C. Then, formazan crystals were solubilized with 100 µL of 10% SDS per well. The absorbance of each well was measured at 570 nm with a Multiskan microplate reader (USA). Each assay, made in duplicate, included a control-containing culture medium. The cell viability percentage was obtained using Equation (3):

Cell viability % = (Absorbance of treated cells/Absorbance of untreated cells) ∗ 100(3)

#### 3.4.2. Antibacterial Activity

The antibacterial activity of Ag_2_Se NPs against several species of bacteria was investigated using a disc diffusion assay. First, 100 μL of bacteria were plated on an LB plate to obtain a bacterial lawn. Then, filter paper discs were dipped into colloidal Ag_2_Se NPs at different concentrations of lyophilized Ag_2_Se NPs: 0 (control), 25, 50 and 100 µg mL^−1^, before being placed on the Petri dishes that contained the bacterial lawns and incubated overnight at 37 °C. The pathogenic bacteria *Escherichia coli*, *Staphylococcus aureus*, *Salmonella typhimurium* and *Pseudomonas aeruginosa* were used as model strains. On the other hand, 1% wt/v penicillin/streptomycin solution was used as a reference drug. Finally, the maximum zone of inhibition, expressed as diameter in mm, was measured.

## 4. Conclusions

The ultrasound-assisted aqueous green synthesis of silver selenide nanoparticles was successful and demonstrated the ability of fructose and starch as biological reducing and stabilizing agents, respectively. The formation of Ag_2_Se NPs was confirmed by a visual color change in the samples after ultrasound irradiation in the presence of both green agents, as well as the UV–Vis spectra with an absorption band in the range of 385–446 nm. The absorbance intensity was observed to be a function of fructose concentration. In addition to obtaining very stable samples, the band gap of these Ag_2_Se NPs is impacted by their size and crystallographic phase. Nonetheless, the fructose amount does not seem to influence their band gap. The XRD patterns showed the presence of both orthorhombic and cubic phases of silver selenide, as well as some prominent peaks that suggest the presence of both elemental silver and selenium. The crystallite size was comparable to the NP size that was determined from TEM micrographs displaying NPs ranging from 5 to 40 nm. Moreover, TEM images confirmed the production of uniform, well-dispersed and spherical Ag_2_Se NPs only in the presence of both natural agents, fructose and starch. The SEM–EDS elemental analysis corroborated the previous results. In addition to C and O peaks due mainly to starch that acted as the capping agent, the EDS spectrum showed the two prominent elemental L-α peaks related to silver and selenium in a 2:1 ratio, confirming Ag_2_Se NP formation. Green-synthesized silver selenide nanoparticles exhibited a high cytotoxic response to the ATCC human fibroblast cell line and a concentration-dependent bactericidal activity against *E. coli*, *S. aureus, P. aeruginosa* and *P. typhimurium*. Overall, our findings’ novelty resides in the efficacy of widespread, renewable and cheap biomolecules to promote the ultrasound-assisted aqueous synthesis of functional Ag_2_Se NPs via a one-step, easy and time-efficient methodology whose versatility may benefit the fabrication of a wide range of nanomaterials of unique features and promising bio-applications.

## Figures and Tables

**Table 1:** Maximum zone of inhibition induced by Ag_2_Se NP samples calculated (in mm) for Gram − and Gram + bacteria.

Strain	Maximum Zone of Inhibition (mm)			
	E_1_	E_2_	E_3_	Ref. Drug
*E. coli* (Gram −)	3.2	4.4	3.4	5.0
*S. typhimurium* (Gram −)	2.0	1.0	3.0	6.0
*P. aeruginosa* (Gram −)	0.5	0.0	1.0	3.0
*S. aureus* (Gram +)	2.0	4.0	5.1	8.0

**Table 2:** Reaction parameters to produce Ag_2_Se NPs in a 100 mL reaction volume.

N°	Composition	V(DIW)	S	F	V(Ag^+^)	V(Se^4+^)	Time
		mL	mg mL^−1^	mg mL^−1^	mL	mL	min
E_1_	AgNO_3_ + H_2_SeO_3_ + F + S	85	10	5	10	5	30
E_2_	AgNO_3_ + H_2_SeO_3_ + F + S	85	10	10	10	5	30
E_3_	AgNO_3_ + H_2_SeO_3_ + F+ S	85	10	20	10	5	30
E_4_	AgNO_3_ + H_2_SeO_3_ + F+ S	85	10	40	10	5	30
C_1_	AgNO_3_ + H_2_SeO_3_	85	0	0	10	5	30
C_2_	AgNO_3_ + H_2_SeO_3_ + S	85	10	0	10	5	30
C_3_	AgNO_3_ + H_2_SeO_3_ + F	85	0	40	10	5	30
